# Correction: Exploring contextual adaptations in caregiver interventions for families raising children with developmental disabilities

**DOI:** 10.1371/journal.pone.0295831

**Published:** 2023-12-08

**Authors:** Zsofia Szlamka, Charlotte Hanlon, Bethlehem Tekola, Laura Pacione, Erica Salomone, WHO CST Team, Chiara Servili, Rosa A. Hoekstra

There are errors in the Funding statement. The correct Funding statement is as follows: This study was part of ZS’s PhD project. It was funded by the Medical Research Council’s Doctoral Training Programme (Funder reference number: 1658511) in the United Kingdom. Her attendance at the WHO CST Consultation Meeting in Xiamen, China, was funded by Autism Speaks. RH, BT, and CH received support from the National Institute for Health and Care Research (NIHR) for the SPARK project (NIHR200842) using UK aid from the UK Government. CH receives further NIHR support through the NIHR Global Health Research Group on Homelessness and Mental Health in Africa (HOPE; NIHR134325) using UK aid from the UK Government. CH also receives support from WT grants 222154/Z20/Z and 223615/Z/21/Z. The views expressed in this publication are those of the authors and not necessarily those of the NIHR or the Department of Health and Social Care. The funders had no role in study design, data collection and analysis, the decision to publish, or the preparation of the manuscript.
