# Supplementary material for: Exploring contextual adaptations in caregiver interventions for families raising children with developmental disabilities
Source: PLoS One. 2022 Sep 28;17(9):e0272077. doi: 10.1371/journal.pone.0272077 (PMC9518887; doi:10.1371/journal.pone.0272077)
Supplement: S1 File — (DOCX) [file pone.0272077.s001.docx]

**S1 File. The WHO approach to adapting the CST programme**

To inform adaptations to the WHO CST programme tested in countries across the world, an adaptation guide was developed by WHO as part of the suite of programme, training, and planning materials to be used by local teams. Programme materials were developed with the aim of applicability to a global audience by using plain language whenever possible, reducing Western biases, such as towards consumerism and individualism (e.g. by almost exclusively suggesting the use of common household items as toys); selecting examples and scenarios with universal applicability, and creating illustrated stories of people of diverse racial and cultural backgrounds in a variety of socio-economic situations and contexts (geographically diverse, rural/urban and in various family structures). To accommodate caregivers with a variety of literacy levels, caregiver booklets that were designed to be culturally and contextually adapted are provided at each group session with illustrated key messages, skills, and strategies to practice at home.

The adaptation guidance for the WHO CST programme was developed using Bernal et al.’s ecological validity model (41), that has been used to adapt parent–child interaction therapy (47). The objectives and process of adaptation of CST were outlined in detail using the Bernal Framework, a method for coding adaptation of interventions (43). The adaptation guidance recommends aspects that should be adapted including (i) translation into the local language, ensuring vocabulary, phrasing and verbal style are culturally appropriate, literacy level is consistent with that of the intended participants, and technical terms are explained appropriately; (ii) adapting the content of facilitator guides and participant booklets (e.g., ensuring names of characters, stories, examples, idioms used in the facilitator guides and participant booklets are appropriate) and process (e.g. location, timing and frequency of group sessions, adding culturally appropriate group opening and closing rituals). Optional adaptations include aspects to improve feasibility and accessibility, including providing childcare, refreshments, or culturally appropriate additional activities. Guidance is also provided on core elements of the intervention that are thought to be necessary and should not be changed (e.g. number and length of group sessions, inclusion of home visits, expectation that caregivers practice skills at home with their child). The adaptation guidance provided with CST also delineates a recommended adaptation process based on the work of Wild (42). The suggested process includes creation of a local adaptation team, formal consultation with an adaptation advisory group of community stakeholders, including caregivers and local cultural and religious leaders where appropriate. The recommended adaptation process is iterative, with adaptation suggested to occur prior to implementation of the programme, and after collecting feedback from participants and facilitators after the first implementation (field test) and after pilot testing.
