# Supplementary material for: Exploring contextual adaptations in caregiver interventions for families raising children with developmental disabilities
Source: PLoS One. 2022 Sep 28;17(9):e0272077. doi: 10.1371/journal.pone.0272077 (PMC9518887; doi:10.1371/journal.pone.0272077)
Supplement: S2 File — (DOCX) [file pone.0272077.s002.docx]

**S2 File. Focus Group Discussion Topic Guide**

WHO CST meeting in Xiamen, China; 8-9^th^ November 2018

This focus group discussion is planned to have an initial, exploratory part of a main project investigating cultural relativism in the planning and design of global child mental health interventions.

**Structure of the focus group and main questions to investigate:**

*Before discussions begin, a brief summary is given to participants about what a PhD project is, what the goals of this specific PhD are and what role the focus group discussion plays in achieving these goals. Prior to the discussion start the principle of confidentiality will be introduced, and all participants will be asked not to share externally what is discussed in the group.*

**A draft welcoming message:**

I am Zsófia Szlamka, PhD student in King's College London in the United Kingdom. My work is supervised by Dr Rosa Hoekstra, who is here today with us, and Dr Charlotte Hanlon. Thank you very much in advance for your time and help with this project.

Can we do a brief introductory round by everybody telling their name, country and professional background? I will start: I am Zsófia, Hungary, PhD student.

As for my PhD, I am interested in the different ways in which people are involved in the implementation of global child mental health interventions, specifically in that of CST, think about what this implementation might look like and to what extent cultural differences and local contexts need to be taken into account in this implementation process. In this study, as we are looking at the cultural adaptations of CST, WHO is a partner and they helped us make this study happen here during the WHO Consultation Meeting.

First, let me briefly describe what will happen in the next hour and how we will do it and then there is a little bit of paperwork to do before we start.

The focus group discussion will last for approximately an hour. I will be asking some questions and we will be conversing about them. I am interested in what each and every of you think about them. There is no right or wrong answer, I am truly interested in all the different views we may have.

This discussion is entirely confidential, please do not share any information other colleagues may have shared in the group with anyone outside this group. This confidentiality will allow all of us to feel confident and share experiences freely.

Participation in entirely voluntary and you share only what you feel comfortable sharing. If you need to leave the room, please let me know.

If everybody consents, he discussion will be audio-recorded. However, once the discussion is transcribed and the data is analysed, original records will be deleted.

Once the discussion is finished, I will transcribe it and anonymise all the data – anonymity is a crucial part of this research. However, many of us may have met before this meeting, and we are all present while our perspectives are being shared. This means that although all personal information will be removed from transcripts and will not be included in any publications following form this research, we cannot completely rule out that you may be identified. The transcribed data will then be analysed to identify the key themes discussed.

Earlier today or over email, when you signed up to the study, you were given a Participant Information Sheet. Has everybody managed to read it or would you like some additional time for reading? Do you have any questions about it?

If you are happy with the Information Sheet, you can now turn to the Informed Consent Form in front of you. Please read it carefully and sign the form and tick the all of the boxes on the consent form if you agree with the points listed.

Do you have any questions?

Please now turn to the Pre-Focus Group survey. Look at me in the eyes when finished, this is how I will now that you are ready to start.

**Warm-up question**

Imagine you are in charge of adapting an intervention. This intervention is targeting children with developmental disorders and it was originally developed in another country. Are there any cultural factors of an intervention that you think are important to adapt from one setting to another?

**1. Cultural relativism in the adaptation**

- To what extent do you think cultural differences may have an impact on the way in which interventions targeting developmental disorders may work?
- To what extent would these occur in different environments, such as a rural or urban setting? Why? What are those differences?
- Whose responsibility is it to ensure that local adaptation occurs? Which groups or who could or should be involved in this work? Who do you think should be investing financially in adaptation?

**2. Universality of evidence**

- You may have heard of the term evidence-base here and there. What comes to your mind when you hear evidence-base? What does evidence-base mean to you? *(addressed first to participants from non-academic backgrounds; doing so by using the pre-focus group survey)*
- Where do you think the balance lies between keeping materials close to the evidence-based original and having local variations?
- Imagine the following scenario: a research group produces some research evidence in a country. What do you think about its application across countries? How do we know if it works? How does this apply to CST?

**3. The adaptation process of CST**

- Let’s first talk about what you as team members experienced during the adaptation process. Were you surprised by anything you learned from the adaptation process?

First let’s talk about any major points that you thought were important to change. Okay, now let’s talk about any minor points you found important to change.

Can you describe any practices of local adaptations that worked well for you?

- Can you describe what was changed after discussions with stakeholders?
- Can you talk about the way in which you discussed these changes with stakeholders and with WHO?
- There are adaptations that are not recommended by WHO. Can you describe if there were any changes that people recommended but you decided not to use? Who discouraged them? Do you know why?

**Closing the focus group**

Is there anything about cultural adaptations and adapting CST that we have missed in our discussion and you think it important? Please tell me about that.
