# Supplementary material for: Exploring contextual adaptations in caregiver interventions for families raising children with developmental disabilities
Source: PLoS One. 2022 Sep 28;17(9):e0272077. doi: 10.1371/journal.pone.0272077 (PMC9518887; doi:10.1371/journal.pone.0272077)
Supplement: S3 File — (DOCX) [file pone.0272077.s003.docx]

**S3 File. Individual interview guideline**

Interviews are conducted in order to further elaborate on questions of cultural adaptation.

**Structure of the interview and main questions to investigate:**

*Before the interview begins, a brief summary is given to the participant about what a PhD project is, what the goals of this specific PhD are and what role the interview plays in achieving these goals. Prior to the discussion’s start the principles of anonymity and confidentiality will be introduced.*

**A draft welcoming message:**

I am Zsófia Szlamka, PhD student in King's College London in the United Kingdom. My work is supervised by Dr Rosa Hoekstra and Dr Charlotte Hanlon. Thank you very much in advance for your time and help with this project.

As for my PhD, I am interested in the different ways in which people are involved in the implementation of global child mental health interventions, specifically for CST, thinking about what this implementation might look like and to what extent cultural differences and local contexts need to be taken into account in this implementation process. In this study, as we are looking at the cultural adaptations of CST, WHO is a partner and they helped us facilitate two focus group discussions during the WHO CST Consultation meeting in Xiamen, China on 8-9^th^ of November, 2018. The focus group discussions gave us some valuable insights and we would now like to explore these insights in more detail through in depth individual interviews.

First, let me briefly describe what will happen in the next hour and how we will do it and then there is a little bit of paperwork to do before we start.

The interview will last for approximately an hour. I will be asking some questions and I am interested in what you think about them. There is no right or wrong answer, I am truly interested in all the different thoughts you may have.

Participation in entirely voluntary and you should share only what you feel comfortable sharing. If you need to leave the room, please let me know.

Only if you consent will the discussion be audio-recorded. Once the discussion is transcribed and the data is analysed, the original record will be deleted.

Once the discussion is finished, I will transcribe it and anonymise all the data as much as possible: I will remove all personal details and country names will be replaced by names of WHO regions. Only anonymous quotes will be used from the anonymised transcripts in publications, however, it might still happen that a colleague identifies the interviewee based on the content of the quote. The transcribed data will then be analysed to identify the key themes discussed.

Before the interview you were sent a Participant Information Sheet. Do you have any questions about it? Would you like to have more time to read it?

If you are happy with the Information Sheet, you can now turn to the Informed Consent Form in front of you. Please read it carefully and sign the form and tick all of the boxes on the consent form if you agree with the points listed.

Do you have any questions?

Please now turn to the Pre-Interview Survey.

**Warm-up question**

Please think about your experience of working with CST.

How did you get involved in CST?

What brought you to the field of autism and to the field of adaptation of CST?

**1. Cultural relativism in the adaptation**

The following questions will be about cultural adaptation and CST. We might have discussed some of these during the focus groups, but now I am specifically interested in your views and experiences.

- At what stage of the adaptation process did you get involved in the adaptation work?
- Are there other interventions you are working with? Can you tell me about them?
- What do you think the parents you are working with need to help their child?
- Can you tell me about the decision to adapt CST in your setting? Why did you choose CST compared to other interventions you may have already used?
- What do you think, to what extent can culture and cultural differences impact whether interventions work? And on whether caregivers are happy to take part in the programme?
- Can you talk about whether the location (geographical region within the country) of where the intervention (CST or others you are working with) takes place might have an impact on whether the intervention works?
- In your setting, who initiated the local adaptation of CST? Which stakeholder groups are involved in this work?
- Does the work get any financial support from any stakeholder groups?
- To what extent do you think the source of funding is important in the adaptation work?

Why is it so, can you elaborate on your thoughts in this regard?

- Can you talk about how these funders were involved in the adaptation work?

**2. Understandings of empowerment and evidence**

What does empowerment mean to you?

What is the outcome of empowerment?

- You may have heard of the term ‘evidence-base’ here and there. What do you expect from a programme that you hear is evidence-based?
- When you started working on CST, how did you think about the evidence it is based on?
- Where do you think we should focus more: on keeping materials close to the evidence-based original or to have variations?

What do you think about the funding of the programme, should it be free or charged for?

Can you tell me about how negotiations go with local authorities/stakeholder groups you work with/WHO/Autism Speaks?

Can you give an example when communication worked well?

Can you give an example when communication didn’t work that well?

**3. The adaptation process of CST**

Can you tell me about how meeting usually go with your team?

Can you tell me about discussions or consultations you may have with WHO, Autism Speaks or other stakeholders?

- Let’s first talk about what you as a team member experienced during the adaptation process. Were you surprised by anything you learned from the adaptation process?

First let’s talk about any major points that you thought were important to change. Okay, now let’s talk about any minor points you found important to change.

Can you describe any practices of local adaptations that worked well for you?

Did you find any practices you originally thought would not work but eventually they worked just fine? Were your original fear unjustified?

- Can you describe what was changed after discussions with stakeholders?
- Can you talk about the way in which you discussed these changes with stakeholders and with WHO?
- There are adaptations that are not recommended by WHO. Can you describe if there were any changes that someone recommended but you decided not to use? Can you tell me what the recommendation was? Can you tell me why it was discouraged?

**Closing the interview**

Is there anything about cultural adaptations and adapting CST that we have missed in our discussion and you think is important? Please tell me about that.
