# Supplementary material for: Exploring contextual adaptations in caregiver interventions for families raising children with developmental disabilities
Source: PLoS One. 2022 Sep 28;17(9):e0272077. doi: 10.1371/journal.pone.0272077 (PMC9518887; doi:10.1371/journal.pone.0272077)
Supplement: S4 File — (DOCX) [file pone.0272077.s004.docx]

**S4 File. Interview guide for WHO and Autism Speaks representatives**

**Introduction**

Can you tell me a bit about yourself? [How did you get involved in WHO/Autism Speaks? What brought you to the field of autism/developmental disorders and CST?]

How did you get involved in CST? At what stage was CST developed when you joined the team?

Can you tell me about how and why CST was started? How was the idea to develop CST born?

Are there any other related or similar programmes you are working on?

How do you see CST in light of the broader strategies of WHO/Autism Speaks?

From your perspective, what are the main aims of CST?

When talking to different teams, something I learnt is that different settings emphasise different aspects of the programme. What is your view on the decision of different country teams to emphasise different aspects of the programme?

How do you see the role of AS to shape how CST is being developed?

And that of WHO?

How do you see the role of country teams?

How do you see the role of caregivers?

There are many stakeholders involved in this work and I’m trying to understand how so many teams and people with different backgrounds communicate or make decisions about the work. What is your experience with this?

Can you give me an example when communication across stakeholder groups worked well?

Can you give me an example when it didn’t work well?

During the follow-up interviews, we talked about **international collaborations** across these different stakeholders. What do you see in the international collaboration that works very well? What do you see to be challenging?

What does this collaboration look like from your perspective? Are there any key issues that need to be addressed?

If you have any concerns about CST, how can you share and discuss it?

**Topics from interviews**

There were a few topics that regularly came up during the focus group discussions or later during the follow-up interviews and I’m interested in your views about them.

You have a broad overview on CST across teams. In your experience, are there any common issues country teams are facing?

If they have any issues, who do they get in touch with?

**Cultural adaptations**: We talked about the extent to which culture has an impact on whether the intervention works.

What is your experience with negotiating cultural adaptations WHO/AS does or does not recommend before the implementation by the country teams?

What is your experience with adaptations country teams come up with?

We also talked about **different sources of funding** that might help or hinder the work. To what extent do you think the source of funding, who gives the funding, is important for the CST work in particular country settings? What do you think is an ideal funding model? Is there any particular model you think is not ideal?

From another perspective, some would argue that caregivers should participate in CST free of charge, while others think it there should be a fee for them to attend. What is your view in this regard?

**Empowerment** came up many times in discussions.

Some participants thought it is a key goal of their work in their setting, whole others set it is not what CST is aiming at. What do you think about this?

I also learnt that there are different ways in which experts, organisations, teams interpret empowerment. What does empowerment mean to you?

**Evidence** and data underlying an intervention was another important topic.

CST is promoted as an evidence-informed programme. Can you tell me how you see the evidence CST is based on?

Some teams mentioned that evidence can be used as a tool to convince other stakeholders to use the intervention. [*For example in countries where there are different parent-mediated interventions available, evidence might be a tool to convince caregivers to choose CST. Another example is that evidence can be used to convince funders to help run the programme.*] What is your experience with using evidence for similar reasons?

Is there something I missed but you feel is important to mention about CST and about cultural adaptations?
