# Supplementary material for: Exploring contextual adaptations in caregiver interventions for families raising children with developmental disabilities
Source: PLoS One. 2022 Sep 28;17(9):e0272077. doi: 10.1371/journal.pone.0272077 (PMC9518887; doi:10.1371/journal.pone.0272077)
Supplement: S1 Table — (DOCX) [file pone.0272077.s005.docx]

**S1 Table. Exemplar quotes**

This document contains the list of quotes relevant to the manuscript titled *‘Perspectives on the role of context in adapting caregiver interventions for children with developmental disorders. Adaptation of caregiver interventions’*

| **Setting the scene for adaptations** |  |  |
| --- | --- | --- |
|  | *PCP128, researcher, AFRICA* | *“I think there is two aspects of culture, the one is what they call the surface level of cultural adaptations so that’s when we changes names and pictures and all of that to make it more acceptable and representative of the culture. But on a deeper level, the cultural beliefs in terms of gender roles, how child rearing practices, so if that is different to the intervention’s core mechanisms of what they are trying to actually achieve, then it’s gonna [going to] influence effectiveness.”* |
|  | *PCP2, caregiver and NGO representative, Americas* | *“We were talking another day that disability is a gender problem because most of the planet are the women, mothers [in charge of the] in care of the children…In our setting, there are groups that are for fathers…sometimes a man does not want to open his heart before another woman…and when this groups of fathers started, it’s a multiplying effect with other men and that is very important because if not, it’s a problem of women.”* |
| ***Cultural adaptation: universal or local?*** |  |  |
|  | *PCP202, clinician and researcher, Americas* | *“For sure the answer is yes [culture is important], but I think also an important thing to consider is how different are the cultures in which the intervention was developed and the intervention that you want to implement…I would think that in our context we are pretty much occidental so in general interventions…developed in Europe may be easy to get adapted.”* |
|  | *PCP6, clinician, Western Pacific* | *“I think to a very small extent that the culture is actually having a specific effect because what’s been discussed in the CST, I mean it is not about culture, it is universal.”* |
|  | *PCP121, caregiver and advocate, Americas* | *“It [cultural adaptation] depends on how your beliefs are, and beliefs is culture. It depends on how your family works, and your family works depending on culture. So I think that the starting point [for adaptations] always is the culture of the region.”* |
|  | *PCP123, clinician, South-East Asia* | *“You can change the names, the pictures or whatever but what’s important is to make those concepts [key strategies in the intervention] relate to the families.”* |
|  | *PCP5, clinician, South-East Asia* | *“There is something that they [caregivers] find very funny, to go on the floor and play with your child, engage with them with toys that don’t have any academic inclination…the idea to just be silly and have fun is somehow culturally alien. So what I say is, the child is just left with himself in big families, so it’s very hard for us to deal with that.”* |
|  | *PCP19, trainer of master trainers, Eastern Mediterranean Region* | *“Culture itself has no impact whatsoever, I think. Because I mean, so basically the work of this [intervention] is between the mother and the child, so…basically the child himself or herself do not have real attachment to their culture, the right thing for me to be adapted, is not cultural adaptation, it is more of the beneficiaries themselves [caregivers], what they are going through.”* |
|  | *PCP128, researcher, Africa* | *“There’s one picture where the clinician tells the mother and the father about the diagnosis…and in our setting they said that no, it will just be the mother sitting there, because fathers are mostly absent anyways in our setting.”* |
|  | *PCP8, researcher, South-East Asia* | *“Different countries run CST program differently and they will implement a lot of their country-specific or culture-specific CST programme, there’s culture-specific elements into the CST programme. So overall thinking, I will have a question, what is CST then.”* |
|  | *PCP21, international organisation representative* | *“Obviously they [adaptation teams] don’t realise the rationale for all elements of the programme so when you explain it, they may reconsider the need for adaptation because they appreciate the rationale for the inclusion of something.”* |
|  | *PCP21, international organisation representative* | *“I think first of all the programme [CST] was developed to kind of minimise the need for cultural and contextual adaptation, that was removing you know biases towards consumerism, individualism, we tried to give examples from the broader world, from different contexts to reduce need for adaptations, that was our goal.”* |
|  | *PCP18, interventionist, Africa* | *“It was kind of difficult sometimes, dealing with children who have different disabilities, so when you’re training on a specific skill, like handwashing skill or dressing skill, not all children will be able to conform so for parents who had children with cerebral palsy as compared to a parent of a child with ADHD or autism, it was very different. So it was a challenge having parents with different disabilities because their abilities were different.”* |
|  | *PCP12, researcher, Africa* | *“The whole idea, the concept of playing with your child, we have discussions of how well that fits with our culture and the communities we want to do CST with. It’s not the principle that might not be acceptable, it’s more the medium, playing materials and toys and all of that, the home routines and the everyday activities, it’s easier to see how we would get a good cultural fit with those activities.”* |
|  | *PCP21, international organisation representative* | *“Just cultural adaptation is not adequate, because for example it might be the same culture in a country with a large urban setting and a rural population, but the context is very different.”* |
|  | *PCP24, international organisation representative* | *“I think the adaptation helps all the stakeholders to get families to be advocates for the programme.”* |
| ***Expectations and beliefs*** |  |  |
|  | *PCP14, caregiver and NGO representative, Americas* | *“So my experience is that they [caregivers] are waiting [for] something, this is assistance mindset, they want somebody to help them.”* |
|  | *PCP9, researcher, South-East Asia* | *“In our culture the parents are looking for some programmes that can be done quickly and efficiently, what I mean is they wanted for the training packs to be quick, and the result or the effect of the training has to be obvious, has to be prominent.”* |
|  | *PCP8, researcher, Western Pacific* | *“I guess it’s the different lens of doing science differently, to me it’s a little bit shock. Because if they [a country team] view this programme as a kind of programme evaluation then they will concern a little bit more about how many participants will be able to join…but to me, as a scientist, I would like to pay much attention on the efficacy of the programme. If you have bias starting from the data collection, even though the result could be very positive or if you can train lots of participants to join the programme, it is not that really meaningful…they view these issues differently, to be honest…and the reason why they would do the other way round is because this person is funded by this agency, so they need to match with the timeline.”* |
|  | *PCP17, clinician, Europe* | *“In our country they [caregivers] think that if you make ABA with the child, you cure the child.”* |
|  | *PCP12, researcher, Africa* | *“If people here hear something like CST, they want help immediately. We are not able to provide help immediately, it’s a long-term project, so that’s difficult to communicate what we want to do and what we can do. That has been a challenge to set realistic expectations and then to communicate that clearly.”* |
|  | *PCP20, clinician, Western Pacific* | *“I think [what families need] it’s the, because the emotional regulation I think it’s quite difficult for children with autism. And actually it’s the biggest challenge in behaviour, for them to adapt to the community, adapt to the school, adapt to their family events. So I think this is the biggest part that the parents concern.”* |
|  | *PCP11, researcher, Americas* | *“We asked the topics to the parents what they want to develop in the project [caregiver intervention]. And they were particularly interested in communication. Their barriers are primarily communication, this is the main thing they would like to develop with their children so that they can understand them, and everything else comes after.”* |
|  | *PCP7, clinician, South-East Asia* | *“Parents are expecting…they wanted something to, I would describe as quite magical to happen.”* |
|  | *PCP24, international organisation representative* | *“Another thing is stigma: changes like the place, in one of the countries, they conducted [the programme] at schools because they couldn’t do it at the clinics because of the stigma.”* |
| ***How to find and engage caregivers*** |  |  |
|  | *PCP19, trainer of master trainers, Eastern Mediterranean Region* | *“During the adaptation we did talk about giving incentives to the parents to come, when we say incentive, we mean the transportation cost to come to the centre where we are doing the training, I mean they are refugees at the end, so the transportation is a cost to them and we do want them to commit because if you don’t commit to the programme it’s a mess. “* |
| ***The role of context and socio-economic factors*** |  |  |
|  | PCP123, clinician, South-East Asia | *“I think because in our setting there is so little available, that the content is very welcome. There is no question everybody [caregivers] wants the content, they want to know more how to help the child.”* |
|  | *PCP1, clinician, Americas* | *“For us [an NGO working with developmental disorders] it’s very important to give specialist advice or consultation to families which are in very remote places and people are very happy with this kind of help, because some people can not travel to the big cities to make to see specialists, so this is a way of really jumping distances and jumping sometimes barriers associated to economic situations…”* |
|  | *PCP205, caregiver and NGO representative, Americas* | *“So short videos, just reminders [would be helpful for caregivers]. Because I think people won’t read the material. I think people in our setting read less than a book per year. So they won’t engage with many pages.”* |
|  | *PCP6, clinician, Western Pacific* | *“Some of the terms, like joint engagement, shared engagement, how we are going to, or maybe home routine, how are we going to translate it to our language and we also need to consider from the layman point of view.”* |
|  | *PCP13, clinician, Americas* | *“Our country is a very big country and we have a lot of difference from the North-East to the South-East and the south part of the country.”* |
|  | *PCP24, international organisation representative* | *“In some countries they couldn’t do home visits because it was dangerous for the facilitators to go into that neighbourhood because it was a high risk neighbourhood.”* |
|  | *PCP5, clinician, Western Pacific* | *“We are working in one of the largest metropolitan areas of our country. So we see various kinds of families, families who are from the metropolitan part of the city, who come for a diagnosis and there are families who may choose to get private therapy services…the third part of families are outside of the city where they come to us to get opinion or they are stuck and don’t receive services.”* |
|  | *PCP16, clinician, Americas* | *“Ours is a big city, how people get to the place, we are in a big economic crisis, so people are very vulnerable, always difficult for them to get here.”* |
| **Technical aspects of adaptations** |  |  |
| **Accessible language** |  |  |
|  | *PCP17, clinician, Europe* | *“In our country [translation]… it is not about the format, there are some words we tried to adapt to make people understand…for example there was noodles, we don’t eat noodles, only some families who go to the Chinese food but not everybody go…yes and in general pasta.”* |
|  | *PCP24, international organisation representative* | *“You know translation has been a huge problem because you know we got the countries…and these countries don’t have money... translation is really expensive.”* |
|  | *PCP20, clinician, Western Pacific* | *“He [a caregiver participating in CST] can speak the Mandarin, he can recognise some characters of the Mandarin. But however, we found that actually his listening of the Mandarin is still not like the native speaker. So because it’s a kind of like barrier to attend this group.”* |
|  | *PCP101, clinician, Americas* | *“Sometimes [caregivers] not even understanding what it meant. So we have a very particular way of using the second person for example, different from all Spanish-speaking countries and so it’s very strange if they would be speaking in normal Spanish.”* |
|  | *PCP205, caregiver and NGO representative, Americas* | *“Because something can have a meaning in the south [of the country] and a totally different meaning in the north of the country.”* |
|  | *PCP19, trainer of master trainers, Eastern Mediterranean Region* | *“I also speak three languages, but when we went to this country [to do the training], I mean you understand with time, but especially when you are working with coaching the master trainers with kids, when they are working with kids, you can’t really know what’s happening because we are not gonna [going to] have a translator for everything.”* |
|  | *PCP2, caregiver and advocate, Americas* | *“Many of these parents are semi-analphabet [illiterate] so you have to write in easy reading…Because that parent has to have a material he can relate to and not just look at the picture.”* |
|  | *PCP6, clinician, Western Pacific* | *“So our parents they are not educated in, you know providing intervention for ASD, so we have to find a term that parents can understand. So mostly about the terminology and the name of the characters.”* |
|  | *PCP11, researcher, Americas* | *“The text and some of the stories were a bit too much for what is possible, because if CST is implemented on a national level, we would have big difficulties with the level of the training and the level of education…we simplified the concepts a bit, but we didn’t want to change the text completely...”* |
| ***Having culturally representative illustrations*** |  |  |
|  | *PCP9, researcher, South-East Asia* | *“We did think about changing the illustrations of the manuals because the pictures about people are rather international instead of having an Asian background so we were thinking about changing that and then a colleague mentioned that all the illustrations were drawn this way, this makes the CST more international and just to letting people know that people from various countries are facing the same thing as you are, so it, it’s uniting people’s mind together so this is a good thing.”* |
|  | *PCP123, clinician, South-East Asia* | *So people, when they saw those pictures, they said oh but we don’t do this and we don’t sit on tables and we don’t have grandmothers who wear things on their heads. But then, other people said that if we present a programme and we talk about how this has been implemented across the world by different settings, in different cultures, then it makes it more appealing because then people realise oh autism is not just my problem* |
|  | *PCP17, clinician, Europe* | *“We discussed few times to adapt the [CST] materials and try to insert the European images in the text.”* |
|  | *PCP205, caregiver and NGO representative, Americas* | *“For us there are some draws [drawings] that don’t make sense, so for example training to eat. In our setting it doesn’t make sense with a bowl. We use different fork. So I cannot present the family this draw [drawing], because it doesn’t have meaning for us. And in fact it’s supposed not to be on the table.”* |
|  | *PCP12, researcher, Africa* | *“Also the use of illustrations in the [CST] booklets and that it was designed for global audience, so the illustrations is helpful for low literacy families.”* |
|  | *PCP23, international organisation representative* | *“The key pictures are the same because the fact it’s being used elsewhere as well, the other community actually enhances the perception of value.”* |
| ***Using online tools*** |  |  |
|  | *PCP1, clinician, Americas* | *“Now we are training 100 parents in the provinces [on the caregiver intervention Transforming Everyday Moments], free, they come from parents organisations, so we did an online course, we are sending mediums [materials] of work and we give suggestions.”* |
|  | *PCP4, clinician, Americas* | *“We can reach out to every place in the country wherever there is [internet] connection… for us it’s very important to give specialist advice or consultation to families which are in very remote places and people are very happy with this kind of help, because some people cannot travel to the big cities to see specialists, so this is a way of really jumping distances and jumping sometimes barriers associated to economic situations…for us technology is a big tool to make information and knowledge accessible to people to make a more fair and equitable access and more democratic access to information.”* |
|  | *PCP22, international organisation representative* | *“I think it may be hard to keep parents engaged in just an online group, they are just not gonna [going to] physically see each other and we are asking them to do it nine times…if it’s a group that is being done online, my guess is that kids could be at home, depending on the time of the day that we do the groups, so they may be distracted.”* |
| ***Intervention model and delivery techniques*** |  |  |
|  | *PCP121, caregiver and advocate, Americas* | *“Families work a lot, they don’t have just one child on the spectrum, they have other children to attend to, they have their job, their other children, the husband, the supermarket, to cook, to prepare everything, laundry, to care for the payment of the therapist, so when you approach and say okay it’s a new project, people want to know how much time is at stake for certain, because many families will not commit because they say I don’t know if I can stay the whole project.”* |
|  | *PCP12, researcher, Africa* | *“We didn’t realise that we will have to adapt the training model [of CST] so extensively…with our master trainers, we wanted to train experts in the provinces, who are busy people so we need to figure out another level how we can quicker train facilitators.”* |
|  | *PCP25, international organisation representative* | *“They [country team] wanted to just make a video, so it’s actually easier for caregivers or whoever they are training to identify these specific strategies and play back and they actually made a lot of different videos to highlight the cases in the manual.”* |
|  | *PCP123, clinician, South-East Asia* | *“We’ve requested [from WHO] that we can break the first session into two halves and the analysis revealed that people [caregivers] might be agreeable to do longer sessions later, but if you tell them from the beginning that you have to come for two hours, people may not turn up. So they come for a shorter session, find it useful and then slowly...”* |
| ***Embedding the intervention to the health system*** |  |  |
| *DDs in health priorities* |  |  |
|  | PCP18, interventionist, Africa | *“There are some few [caregivers] that were giving really so much priority to these children with disabilities, that they weren’t taking care of themselves and as a family at life, so they weren’t giving involved in the family because they felt they have a child with special [needs], and they have to take care of them on a daily basis. But for some few, they preferred going out to get something for the family as compared to staying with this child, so they would get someone to stay with them or they would finally take them to school, because they were able to do the other daily tasks, so they can be able to fund for the family.”* |
|  | *PCP22, international organisation representative* | *“So […] for autism and developmental disability, the evidence base is thin and it wasn’t a priority for multiple reasons. One is for some countries was the level of awareness, not really knowing anything about it and then from a system perspective, not like in other, chronic conditions or communicable disease where there are maybe limited points of entry to the system of care. With autism, you can come at it from an early childhood development perspective, you can come at it from disability perspective, you can come at it from a mental health perspective.”* |
|  | *PCP22, international organisation representative* | *“There’s this stigma that goes with mental illness in general, anything that falls into that category, and then the stigma specifically related to autism, or other neurodevelopmental disorders where there’s no cure, so why should we devote this money to improve kids who are just not gonna [going to] get appreciably better, I think this is their perspective, devoting it to an area where you can see more visible changes.”* |
| *Low-resource setting and poverty* |  |  |
|  | *PCP14, caregiver and NGO representative, Americas* | *“Some families don’t want the kids to make improvement because they will lose anything they receive from the government…so because it’s a minimum salary because of the handicap of the kid, so they don’t want them to do anything…so there is some challenge that is deeper.”* |
|  | *PCP19, trainer of master trainers, Eastern Mediterranean Region* | *“Usually the priority for them as a family is financial, that is for sure. Getting yourself violently [detached] from your home and from your work and everything, you start feeling that there is no net to fall on in case of anything happens, because you don’t have financial stability, so for sure the financial aspect is their priority.”* |
|  | *PCP23, international organisation representative* | *“A lot of our countries have nothing [for DDs], have zero, no programmes, no data...“* |
|  | *PCP126, caregiver and advocate, Americas* | *“It’s [treatment] really expensive in the private service. In the public, there is very limited number of this kind of treatment. So when they get this, it’s like once a week in the better situation. Sometimes they have to wait like a year, two years, to have access for these treatments.”* |
|  | *PCP126, caregiver and advocate, Americas* | *“There is no faculty, or no university where you could learn about autism, do a doctoral degree, get some licence, nothing.”* |
|  | *PCP6, clinician, South-East Asia* | *“In our country parents are not ignorant about strategies or interventions available for ASD. Some of the parents are quite educated and quite knowledgeable about what kind of strategies are effective for this kind of students or children. So I think it depends on how developed the city is in terms of supporting students or children with SEN [special education needs]...”* |
|  | *PCP10, caregiver and advocate, Americas* | *“A lot of these families, they don’t have the eligibility with autism either to the school system or early intervention, and this [CST] is really for anyone, so if your child is having social communication challenges, this would be a programme for you, and then something that you don’t have to go to a clinic to do, you don’t have to go to the school to do and we really emphasise you know embedding these strategies within home routines.”* |
|  | *PCP11, researcher, Americas* | *“There’s more services in cities compared to rural areas and places farther away, it’s much more difficult there. As for basic education, there are many children who have to walk at least an hour, hour and a half to school, so it’s more or less the same when it comes to accessing programmes.”* |
|  | *PCP121, caregiver and advocate, Americas* | *“I have been also helping in context of favelas [poor districts] so people that speak in a different way and with a lot of my heart [while being compassionate] but are very ignorant, you can not speak in a difficult manner, you have to be frank and direct and easy.”* |
|  | *PCP123, clinician, South-East Asia* | *“I know that they [WHO] had expert reviewers [to review the CST materials], we do not have any copyright issues because they all agreed that these parts of their interventions could be included in in the package.”* |
|  | *PCP11, researcher, Americas* | *“The doctor went to work to another region and so at least there was a psychiatrist in the province. He told us that these community mental health centres in the provinces they were step by step slowly implementing these basic elements and there’s still many things they are not covering, like with so many things, it’s gonna [going to] be quick in the capital and then it will spread in the provinces.”* |
|  | *PCP12, researcher, Africa* | *“When there isn’t a good system and there’s hardly any services, it’s asking different questions compared to a country where there’s existing services. We are realising that it can be a great intervention with very good evidence based on another country but there can be built-in barriers, things like supervision and training and monitoring of therapists and all those extras…”* |
| *Health systems with competing services available* |  |  |
|  | *PCP121, caregiver and advocate, Americas* | *“That happens in our setting with one day trainings, that everybody wants trainings to be free, you have 300 registered people and then you have 50.”* |
|  | *PCP17 clinician, Europe* | *“Only I try to make some advocacy, that look, this is also a programme and if you are interested and it’s a very hard…because they read on the internet about the ABA and it is really a voice in our country.”* |
|  | *PCP4, clinician, Americas* | *“So different experiences, but I think they feed each other, because when the master training was done here, many of the ideas and recommendations that came up to improve the master training came from our experience with the other workshops, you know, how to train facilitators…there were a lot of things that we had learnt from the other workshops that would be able to facilitate or make CST easier. ”* |
|  | *PCP19,* *trainer of master trainers, Eastern Mediterranean Region* | *“In our region, anyone who works with developmental disorders and more specifically with autism spectrum disorder is not free, is always expensive, even costly for the NGO itself because it needs a team that works on a daily basis.”* |
|  | *PCP25, international organisation representative* | *“They [a country team] also noticed that while they are coaching the parents, a lot of them had mental health issues that they brought up, either family or mental health issues, so they created a whole directory of resources for families.”* |
| ***Sustaining the intervention*** |  |  |
| *Financial sustainability* |  |  |
|  | *PCP11, researcher, Americas* | *“The whole initiative [to adapt and implement CST] started in the ministry of health, it’s part of the state’s health strategy, and specifically that of mental health. And so the financing of the adaptation is through Americas…and the implementation of the programme takes part of the community mental health centres…it will be part of the normal functions of these centres…also the facilitators’ training will take place there.”* |
|  | *PCP23, international organisation representative* | *“The cost, the economics of this programme [CST]…nobody is thinking about that. One way in which you pitch to policy makers is cost effectiveness analysis. We don’t have that. But it will be important to have that, you know. Because it even speaks to the whole idea of our intervention, right, because the mantra has been that if you’re able to access the intervention, you have better prognosis and you reduce needs throughout the lifespan.”* |
|  | *PCP22, international organisation representative* | *“That petitioning with the ministry of health, to provide the funding was very challenging, we were not successful, it’s small amount of money that countries are asked, contribute to other more higher priority things.”* |
|  | *PCP15, clinician, Americas* | *“I understand that there are resources, like you know who is gonna [going to] go out there to do the training for the caregivers…they [intervention facilitators] learn the intervention, sure they can continue on their own but there also needs to be a little bit of funding for those who are training the interventionists, but I don’t think it should be like expensive…the whole point of this project that these families can actually access service, access it because they don’t have the money to access it. So it would defeat the purpose if we start charging for this programme.”* |
|  | *PCP9, researcher, Western Pacific* | *“We realise that there’s so many things that we need to buy…for example toys…we have to ask for extra resources from the funders…we had to book rooms for meetings and that counts and we had to do like so many facilitator guide and participant booklet copies in colour…so the budget that we have is quite tight and we had to think carefully of what we need to buy.”* |
|  | *PCP11, researcher, Americas* | *“Sometimes you open a [mental health] centre but there’s a lot of bureaucracy and the lack of funding slows it down and maybe the centre has to provide everything because there’s no funding to pay the psychologists who work there.”* |
|  | *PCP12, researcher, Africa* | *“So I think the one that we’re trying to avoid at this stage is volunteerism, where people are doing the things with CST without getting paid...because it might not be sustainable”* |
|  | PCP5, clinician, South-East Asia | *“The funding status matters because we are approaching other organisations, the first thing they ask is do you have funding for us to do is. And so I mean it’s hard for us, because a lot of investment of time that people have to make and when people ask to be compensated for their time to learn this new programme and implement it and do research.”* |
|  | *PCP3, clinician, Americas* | *“We have been talking to different people, like insurance, companies. We explain what CST is about and that might be interesting to do our research on the benefits of this programme and so on and they said that they were very interested to get involved. But the thing is that, one thing is what people say…but when they have to put the money, that’s another conversation.”* |
|  | *PCP15, clinician, Americas* | *“There is a mother, she wants to help people that don’t have the same situation of her, because she can pay all the treatments for his children, she has good economic situation and she bring she make the contact of WHO. She did this choice by herself, she has an NGO.”* |
|  | *PCP25, international organisation representative* | *“The Asian community is like oh we are able to secure grants so definitely we are able to fund this [CST]. So I think that’s why that specific Asian community started one of the trainings.”* |
|  | *PCP19, trainer of master trainers, Eastern Mediterranean Region* | *“In NGO settings the measurements for quality are not the same as in academia setting, kind of they focus more on quantity and indicators, but in universities they focus more on research, which is that added thing of research.”* |
|  | *PCP24, international organisation representative* | *“The ideal thing would be to have research grants and to have this [CST] as research process and research includes identifying what sources in the community could sustain the programme. But the first step should be research funding, you know. That is the most expensive part of the programme.”* |
|  | *PCP1, clinician, Americas* | *“We tried with many governments and with many ministries to really show the project, we finally got the social development, so we didn’t have luck with health ministry, with mental health office. We knew that there was this kind of funding that they give every year to community projects, so since we wanted to do it [CST and Transforming Everyday Moments] in their community centres, we applied in the same ministry to get the funding, in another office, connected the two offices and said, listen we are doing it for you, the office around the corner.”* |
|  | *PCP25, international organisation representative* | *“I mean they [funders] are all different but I think the ones that are able to get like the health administration, like that kind of funding from state or federal level to try to integrate it to their country, I think the likelihood of sustainability is higher versus getting like grants, because grant is like a one-time funding, versus the others that really try to integrate it to some kind of system.”* |
|  | *PCP23, international organisation representative* | *“I’m not naïve to say that I can absolutely control that no private sector entity can charge for the programme…in the future, I know that’s gonna [going to] happen, right, but I think we should try to do our best we can to make sure that accessing it without a charge is an obligation… but I do think that the private sector can, they can offer this, as a lack of better word, in marketing, as a leader, right, so you offer this as introductory level support service for free and we know that most kids will require additional support in specific areas to build specific skills and then the private sector can charge for the added services, not CST.”* |
|  | *PCP1, clinician, Americas* | *“They [funding organisation] don’t care much about what it is about, only that it’s directed to the community and they send some people to see what you are doing 2-3 times a year…they are not involved in like measuring the impact or know exactly the maybe the technical part.”* |
|  | *PCP6, clinician, Western Pacific* | *“They [funders] just sit there and observe and very rarely they give any opinion or raise any questions. But we know that they fund us, so we got a lot of respect to the observer you know.”* |
|  | *PCP16, clinician, Americas* | *“Last year we were working with the ministry of health to get some funding to make a big training for all regions, the country is very huge, so we needed to get a very big funding to get this, but the government has changed so we don’t think it’s going to work.”* |
|  | *PCP9, researcher, Western Pacific* | *“They [private funding organisation] funded some other NGOs as well, and because they knew that… there were some adaptation going on and so they wanted to get those NGOs whom they are working with to get involved, mainly and because we wanted to get so many comments on the adaptation of the CST. So when funder said to these NGOs that you need to be involved in the CST, than the NGOs had to follow…but the university is urging us to start in three months, so that’s a bit of a big pressure to us.”* |
|  | *PCP22, international organisation representative* | *“Actually how we advise them for higher income countries, they [clinicians] are seeing this as an option particularly for the kids who are on the wait list for more services.”* |
|  | *PCP1, clinician, Americas* | *“It was like necessary to have the government connection and so the community centres, the people who are facilitators we are training, they already had salaries there, so we are sure they will stay there and they will not charge anyone, it’s already a community centre so I think it was a good deal because we know that they will work and they are doing it for free.”* |
|  | *PCP4, clinician, Americas* | *“So a lot of this work has been done on voluntary basis, we’ve been working on this in our own time and…it’s difficult to get money out of the government and it’s difficult to get money out of private parties that are interested in funding these kind of initiatives…in the meantime we still work.”* |
|  | *PCP7, clinician, Western Pacific* | *“Our government…they don’t really want to take up responsibility to deliver many services that requires a lot of money.”* |
|  | *PCP24, international organisation representative* | *“You can tell ministry of health, ministry of education, NGOs, whoever you are working with, to tell the master trainers and facilitators to do this. Because they say yes, we are gonna [going to] collaborate, but when they see what the master trainers need to do…they [employers] want them [intervention facilitators] to do in their own time, so that’s awful.”* |
|  | *PCP19, trainer of master trainers, Eastern Mediterranean Region* | *“It was a change in strategy, they [an international organisation] wanted to fund more education in the refugee situation and less in mental health so that money just went to an education programme.”* |
| *Role of funders* | *PCP7, clinician, Western Pacific* | *“They [the funder] have to contribute several percentage [of their profit] back to society, there’s a law. So it is a very wealthy organisation so that’s why they take up a wide range of our country’s social services and sponsor many of those.”* |
|  | *PCP1, clinician, Americas* | *“The fact that you can do it [a caregiver intervention] for free has the advantage that any family can participate…we are an NGO and we did some things for free for the community…but we can’t afford to everything for free, so this model of getting funding from the government, it worked.* |
|  | *PCP23, international organisation representative* | *“When you connect with the right people, you can dramatically accelerate progress [with advocating for an intervention].”* |
|  | *PCP4, clinician, Americas* | *“It [how an intervention is chosen] has to do with the people you meet in life…it has to do with the networking between researchers. If I had met this other person before and talked about that particular intervention, maybe we would be using that particular intervention in our country, I don’t know!”* |
|  | *PCP205, caregiver and NGO representative, Americas* | *“In our context to have the minister of health they are not, you know, from Earth. They are So far that they don’t know what happens and we need the city resources, so in the ministry of health they have the money for it, but they don’t have the physical and workforce resources, so that’s why we decided to run locally in our setting.”* |
|  | *PCP8, researcher, Western Pacific* | *“I found that…there it’s a kind of a mismatch between the expectations of the funders and also the entire research timeline.”* |
| Availability and commitment of intervention facilitators |  |  |
|  | *PCP127, researcher, Western Pacific* | *“How we are able to sustain the programme for example in ten years’ time. Because some master trainers may not be able to train facilitators later on. Some just want to have the qualification of the of the training of the programme and they might say oh I’m a master trainer but I may not help you to train facilitators, they just want to get the qualification of it.”* |
|  | *PCP18, interventionist, Africa* | *“But for a larger cohort, it will take time [to implement the intervention] because you will need several staff to be able to go for these home visits…because they really needed things to be explained about their child, how they are doing, so I think it needs more manpower, more staff to do these fieldworks, do the home visits and then do the trainings. The training is not so much…”* |
|  | *PCP202, clinician and researcher, Americas* | *“We are thinking of having this [CST] as part of curricula in the school of occupational therapy and infirmary, and that will give us a huge critical amount of people that will be exposed to the intervention. So they may not like it but you can identify those who really like it and they may be more engaged in the future to work on the intervention.”* |
| Managing long-term stakeholder collaborations |  |  |
|  | *PCP11, researcher, Americas* | *“It became impossible to coordinate [different stakeholder groups]. I think for this reason it was very important that the stakeholders from these [different stakeholder] institutions were very motivated and wanted to help. The extended group [of stakeholders], it was much more difficult, so we had to have individual meetings with all the members of all the institutions so that they support us.”* |
|  | *PCP23, international organisation representative* | *“Our strategy usually is top down, bottom up. We work closely with local advocates, we try to establish strong ties with local parent organisations, because they are the real leaders in those communities and they know what’s going on, not like us. The advocates, the families are the ones who go to the government and say we need this programme. Or to find somebody in the government say we need this programme, you have to help me bring it in.”* |
|  | *PCP22, international organisation representative* | *“What we saw in this country, where there are several small people [lay people without positions in institutions of power] and academics working independently and it’s just hard to get the ministry to focus, as a country it’s still even focused on morbidity.”* |
|  | *PCP12, researcher, Africa* | *“It is sometimes challenging keeping up with communication to make sure that everybody is informed the whole time about what’s happening and you might be doing training and you are very involved with the training group and then you realise oh no, you haven’t talked to the stakeholder advisory group and then you realise you haven’t talked to WHO about things.”* |
|  | *PCP24, international organisation representative* | *“In our region we don’t have research culture. So many universities don’t have a good research component, so we have had to help countries to build. But in some countries their process hasn’t advanced because they don’t have a research team to work with.”* |
|  | *PCP6, clinician, Western Pacific* | *“All the master trainers participating in this programme, they are all very, very, very busy, so it’s very hard for us to gather, it’s a miracle for us to, coming from different NGOs and gather in one venue in one time and so on day-to-day basis we don’t have much communication and that is a pity I would say because professionally sharing is quite important.”* |
|  | *PCP11, researcher, Americas* | *“With the professionals involved, there were no issues, for example, the psychologists, psychiatrists, they already participated in a training, so they already had an interest to be involved in this. The training wasn’t an obligatory thing, if you were interested only, so you needed a personal motivation for the process.”* |
|  | *PCP24, international organisation representative* | *“If the process is managed at an upper level, in the ministry of health they decide everything, but they don’t involve the people that are gonna [going to] be the master trainers, that is awful. Because it is like this, the people down the wall of the ministry of health [everyone who is not in the ministry] feel that they weren’t taken into account when they were planning, so also when we have master trainers, the communication is like why do you have to supervise me, if I’m a child psychiatrist, I can do it. We have two or three countries like that and they didn’t advance because all the decisions were taken on an upper level and the implementers weren’t consulted, they just gave them an order. So if you from the day one incorporate all the stakeholders and the decision-making process, you are gonna [going to] have a better odds of success.”* |
|  | *PCP203, clinician and researcher, Americas* | *“The cultural difference…this is the reason why I think that’s important to design the interventions with the local people…they need to say something about…because the local contexts can modify what is the best way to develop the intervention.”* |
|  | *PCP4, clinician, Americas* | *“More controls, and more trying to figure out what is working, why it’s working, and in the other sense, these workshops, they are only under our control, it’s much easier to have more liberty and do. You just do go ahead and do it, we don’t have to give explanations to people and you know, that sometimes, it stops the time.”* |
|  | *PCP12, researcher, Africa* | *“As a more Western society we tend to present written information and might be using Powerpoint format, that’s our preferred way to communicate but that might not be the community’s way to communicate. In more rural stakeholders, who don’t necessarily use emails, that’s not their preferred way of communicating, you need to phone them, it’s more difficult to coordinate and connect the group.”* |
|  | *PCP10, caregiver and advocate, Americas* | *“There are two psychiatrists from the ministry, and some things they said gave me the impression that, because they don’t have children with autism, there are things they don’t know. Because they don’t live up to it, they don’t know. And so it is our task to tell them, for example, my son won’t understand what you say, won’t remember, but if you show a small video, he will recognise that. So, for me it’s important that you don’t just have the material for the pilot, but that is has graphics, it has videos, and so it is a tool for children to understand [the tasks] better, right?”* |
| *Leadership* | *PCP25, international organisation representative* | *“He [a CST country team lead] is so invested and because others can see how invested he is and then he makes sure, like decide how this work will go too, they kind of follow his lead.”* |
|  | *PCP205, caregiver and NGO representative, Americas* | *“I’m in the middle [of managing stakeholders], I talk with the public service, I talk with the researchers and then I have my own thoughts.”* |
|  | *PCP22, international organisation representative* | *“It’s usually a very persistent person within that country that keeps it going, barely it’s the ministry of health say we want a programme and we searched the WHO website and we found this programme, it doesn’t happen. It’s a parent or a professional who says, we want this, we think this would be good not just for us but for other parents and want you to make this happen.”* |
|  | *PCP24, international organisation representative* | *“I believe in this [CST]. It is not that it’s just my job. I believe in the power of transferring the power to the countries.”* |
|  | PCP9, *researcher, Western Pacific* | *“We would just try it [CST] out because it’s greatly recommended by the WHO, so yeah we believed that there must be something that CST is working so well, that WHO is saying this is going to work.”* |
|  | *PCP17, clinician, Europe* | *“I try to involve the representative of the ministry, I asked some official paper [from WHO] to be send to the one of the hospitals to present the programme, and they tell me that is not the job. But in our country we need some official paper, because without this I am nothing…and now I don’t do nothing because I was a little disappointed because of their attitude, because I really like the programme and I really make a lot of things, all these things from all my time.”* |
|  | *PCP12, researcher, Americas* | *“It’s motivating to be part of a global initiative, so it’s definitely helpful to be associated with other countries that are also doing it, it helps our conversations with our government and our organisations to obtain buy-in more easily because they know many people are doing it.”* |
|  |  |  |
