# Supplementary material for: Exploring contextual adaptations in caregiver interventions for families raising children with developmental disabilities
Source: PLoS One. 2022 Sep 28;17(9):e0272077. doi: 10.1371/journal.pone.0272077 (PMC9518887; doi:10.1371/journal.pone.0272077)
Supplement: Attachment — Submitted filename: PLOS one review 19.2.22.docx [file pone.0272077.s006.docx]

Thank you for the opportunity to review this manuscript, entitled, ‘Perspectives on the role of context in adapting caregiver interventions for families raising children with neurodevelopmental disorders’.

This is, in general, a well conceptualised and written paper, methodologically sound and with results that are relevant and meaningful. The discussion is carefully considered and integrates appropriate literature. As a result, I have relatively few comments and suggestions and would like to congratulate the authors on their work in this important area.

**Introduction**

The introduction is concise and addresses the salient areas. I did wonder if it would be useful to add some pieces regarding the risks or challenges of task sharing, particularly in LMIC (which could then link in with the challenges of funding and sustainability in the discussion section later). This aside, I don’t have any other specific suggestions for changes.

**Methods**

I would have liked to know how many people attended the technical consultation meeting in Xiamen, China – 15 accepted the invitation to participate in the focus groups, leaving how many who did not?

Were there cases where participants were both a researcher and a clinician? If so, how was it decided where they were assigned in the table?

It would be useful to know a little more about the participants and their background, specifically their experience with the CST, e.g. were most of them in the starting phase, how many had completed the adaptation process etc. How many worked in NGO’s vs public service etc. While there was an excellent range, if there was a big proportion working in NGO’s for example that would be important to know.

The researchers positionality was thoughtfully described, I would suggest it be important to describe their own background (i.e ZS and RAH), given it’s relevance and potential for bias. E.g. are both researchers or also clinicians, do they hold more than one role and was this shared with the group?

**Results**

The results are well described and concisely articulated. The use of quotes is appropriate and adds to the section.

Perhaps I missed it, but I think it would be helpful to have a brief description/explanation that data from phase 1 and 2 were amalgamated in the results section.

When sharing quotes from participants, individuals were given different descriptions – e.g. ‘clinician’ or ‘caregiver and advocate’, others had more detailed descriptions, e.g. ‘trainer of master trainers’. I would suggest, if possible, more consistency, perhaps one of clinician/researcher/caregiver and then an additional description of the participant (if possible)?

**Discussion**

The discussion was considered, included relevant literature and outlined some of the complexity of this subject area. The authors at times chose to reflect opposing views expressed by the participants which added to the richness of the section. The key sections of the results are elaborated on and examined.

Although the implications section raises salient points, I wonder if there could be a little more on reflections for future research in this area. Given how challenging it is to capture the role of context in adapting and implementing caregiver interventions, providing some guidance in the area would be useful.

Page 25, last paragraph, line 5, perhaps this could be rewritten for clarity.

**Limitations**

I think a line on the ways in which not having stakeholders who left CST participate in the study is a limitation, would be useful.

Is a further limitation of the study that it only included stakeholders directly involved in CST? I would think that gathering information from those not involved in the programme (and outside the structure and framework of WHO) may gather further, and diverse information. Given this was a CST meeting, I also wonder if, as was alluded to, there may not have been some bias, that participants may have felt a need to be largely positive about the ways in which CST was managing adaptations and implementation (e.g. not emphasising those areas it was not managing so well).

**References**

I would suggest a look over the references for consistency, I noted for example #’s 8, 13, 58 and 62
