# Supplementary material for: Exploring contextual adaptations in caregiver interventions for families raising children with developmental disabilities
Source: PLoS One. 2022 Sep 28;17(9):e0272077. doi: 10.1371/journal.pone.0272077 (PMC9518887; doi:10.1371/journal.pone.0272077)
Supplement: Attachment — Submitted filename: Response to Reviewers 30.06.2022.docx [file pone.0272077.s008.docx]

30.06.2022

Re: Response to reviewers of Manuscript [PONE-D-21-29533R1] - [EMID:3cb16f3caf5a7a17]

Title: Exploring contextual adaptations in caregiver interventions for families raising children with developmental disabilities

Dear Dr. Eric J. Moody,

Thank you for your invitation to revise and resubmit our manuscript following the helpful comments from the reviewers. Below we have responded in detail to each of the reviewers’ comments. Reviewer comments are in italicised text and our responses are inserted after each comment in plain text. The revisions are also highlighted in the manuscript text itself using tracked changes.

*Reviewer #1: The authors have made detailed responses to the comments raised by the reviewers. I have only minor suggestions to make.*

*Comment 1. Abstract: 4th last sentence (Participants discussed ..) is unnecessary as it repeats two theme headings without adding information, therefore omit.*

Thank you for the suggestion. We omitted this sentence. The Results section of the Abstract now reads as follows:

Four main themes were developed: 1) Setting the scene for adaptations; 2) Integrating an intervention into local public services; 3) Understanding the reality of caregivers; 4) Challenges of sustaining an intervention. Informants thought that contextual adaptations were key for the intervention to fit in locally, even more so than cultural factors. The socio-economic context of caregivers, including poverty, was highlighted as heavily affecting service access and engagement with the intervention. Competing health priorities other than DDs, financial constraints, and management of long-term collaborations were identified as barriers.

*Comment 2. On page 5 the word 'context' is given in italics. I think it would be helpful to do the same on page 28 for the words 'tension' and 'respect'.*

We took your advice, and the words ‘tension’ and ‘respect’ are now written in italics on page 28. It reads as follows:

Examples include *tension* being a specific idiom of distress in India (56) or *respect* a specific Latin American value of parenting (57).

*Comment 3. Page 6, line 7: An example of (not 'to')*
Thank you for spotting this mistake. We corrected it and the sentence reads as follows:

An example of this cultural focus is using the term ‘ubuntu’ to ground early interventions for DDs in Africa (75).

*Comment 4. Page 8: Table heading has mixed font.*

Based on your comment we made sure that the table heading has a consistent font on Page 8 and it now reads as follows:

**Table 1. Characteristics of participants in the focus group discussions**

*Comment 5. Page 28, line 10: should this be 'joint' (not joined)?*

Thank you for spotting this error. We changed ‘joined’ to ‘joint’ and the sentence now reads as:

For example, a previous autism intervention study in the USA suggested up to 69% of the intervention effect on language improvement may be mediated by joint engagement (74).

*Comment 6. Page 29, line 6: should this be 'benefits'? I think better to say 'receipt of financial benefits'.*

Thank you for the suggestion. We changed ‘benefit’ to ‘the receipt of financial benefits’. The sentence now reads as:

Caregivers may not be informed about DDs (believing that DDs can be cured) and they may not have accurate information about their rights (when would the receipt of financial benefits stop).

*Comment 7. Page 31, Limitations 2nd sentence, line 4: comma needed after 'programme'.*

We added the comma to the second sentence of the Limitation section. It now reads as:

Including voices of those working with different interventions, perhaps also some who may deliberately have chosen a different intervention because they are critical of the CST programme, may have provided more diverse information.

*Reviewer #2: Thank you for comprehensively addressing the comments, I have no additional recommendations and believe it to be an important contribution that will increase our knowledge in this area were it to be published. Again, congratulations on your work.*

Thank you for providing your thoughts and feedback.

We hope these revisions are to the editor’s and reviewers’ satisfaction.

Kind regards on behalf of all authors,

Zsofia Szlamka,

Corresponding author
